# Competitive Golf: How Longer Courses Are Changing Athletes and Their Approach to the Game

**DOI:** 10.3390/nu14091732

**Published:** 2022-04-22

**Authors:** Matthew Zoffer

**Affiliations:** Department of Ob/Gyn, Fort Pierce Regional Campus, Florida State University College of Medicine, Fort Pierce, FL 34981, USA; drzoffer@nutrytics.com

**Keywords:** sports nutrition, golf, distance insight report, PGA

## Abstract

Nutritional guidance for competitive golfers to improve performance is limited. Recommendations and study conclusions from older research used smaller golf courses compared to today and require a reevaluation of energy expenditure. This review identifies aerobic fitness, in addition to strength, as a key determinant of success. A novel nutritional approach that incorporates carbohydrate supplementation to support aerobic fitness without sacrificing the ability to build strength is presented since longer courses require more stamina. Strategies for training, competition, and recovery are outlined based on different skill levels. American College of Sports Medicine (ACSM) guidelines for carbohydrates, protein, and hydration intake are tailored specifically for competitive golf based on this approach. Putting requires precise movement and can be affected by fatigue. Nutritional studies in golf and similar sports that require focused movements are presented, exhibiting an improvement with adequate hydration and carbohydrate status and caffeine use. Competitive golf poses unique challenges to an athlete and commonly used ergogenic supplements that can improve performance in a variety of circumstances during training, competition, and while traveling are reviewed.

## 1. Introduction

Golf has been played as a sport for over 500 years and competitively for over 160 years [1]. In 2021, total purses for the 50 PGA Tour events are worth over USD 392 million. Golf is fiercely competitive at this level, yet there are few peer-reviewed articles focused on nutritional strategies that can improve performance. A literature search for “Golf and Nutrition” in PubMed yielded no studies solely dedicated to the sport, and they all used golfers as part of a larger cohort. Performance improvement articles in the literature focus on strength and mechanics to increase ball distance or assess fitness in casual golfers.

This review will illustrate that walking the course utilizes the majority of energy during a round of golf compared to club transportation and striking the ball. As the physical demand of walking the course increases, so does the oxygen demand, despite golf being played at moderate physical activity levels [2]. The Distance Insight Report jointly authored by the Professional Golfers Association (PGA) and Royal and Ancient (R&A) has concluded that golf courses are longer, yet strategies to train, fuel and supplement these athletes have been lacking in the literature that acknowledges this information. An approach to incorporating aerobic training and complementary nutritional strategies during training, competition, and recovery is not currently recognized as a method to improve performance for competitive golfers.

## 2. Energy Calculation

### 2.1. General Considerations

The cornerstone of performance nutrition is an evaluation of energy expenditure. There are three areas of analysis while playing golf: Club transportation, walking the course, and hitting the golf ball. 

### 2.2. Club Transportation

The United States Golf Association (USGA) allows players 14 clubs in their bag including water, rangefinder, balls, and tees. Older studies measured the weight of golf bags from 8–30 kg (18–30 lbs), but this was prior to synthetic bags or did not include other necessary supplies [3,4]. According to Shipstix which transports golf clubs, “A golf bag equipped with clubs, balls, and accessories averages 20 lbs (9 kg)” [5].

In 2009, the USGA allowed the use of pushcarts as an alternative to carrying the golf bag in high school and collegiate golf tournaments for men and women athletes [6]. In 2019, 67% of all high school golfers used a pushcart in competition (94% girls/52% boys). At the college level, 2014 National Collegiate Athletic Association (NCAA) champion Cameron Wilson and most of his teammates at Stanford, and 50% of the men’s team at UNC used a cart [7]. The energy expenditure comparing the two forms of club transportation are similar. In both the Crowell and Dear studies, carrying clubs for 9 holes was 411kcal and 511 kcal, respectively. While using a pushcart expended 411 kcal in Crowell et al. [3,8]. 

Professional Golf Association (PGA) and Ladies PGA (LPGA) players use a caddy and club carriage should not be part of the energy calculation.

### 2.3. Distance 

The United States Golf Association (USGA) and Royal and Ancient (R&A) mandates that all “Players shall walk at all times during a stipulated round” [6].

The distance walked by a golfer is affected by several factors and is increasing.

Each golf course is uniquely designed, and distance is variable based on gender and skill level by moving the tee box further from the flag. The current distances for the different competitive levels are listed in Table 1 [9,10].

Distance from tee box to flag is not the actual distance walked since golf shot accuracy varies. Golf shot dispersion measures off-center shots when the ball is mishit by the golfer. A shot with a +4° error will finish 14 yds to the right of the target on a 200-yd shot and 21 yds to the right on a 300-yd shot. Golfmetrics data provided by Broadie et al. concluded that higher caliber golfers hit the ball straighter more consistently than less skilled golfers with higher dispersion [11]. The distance walked is reduced as skill improves. The Distance Insight Report, jointly authored by the United States Golf Association (USGA) and the Royal and Ancient (R&A), found that course length is also increasing. Due to improvements in technology and athleticism, the average driving distance by Professional Golf Association (PGA) Tour professionals is now 310 yards. This has increased the total course length by 400 yards since 1980.

The integration of golf courses with residential communities has increased the distance from the green of one hole to the tee box of the following hole and increased the total course footprint [12]. The USGA used aerial photography and compared an 80-course random sample of USGA-approved courses, including 15 championship courses. “Courses built during the 3 most recent decades had an average total footprint of 216.3 acres. Those built during the 1920’s through 1940’s had an average footprint of 152.3 acres a difference of 64 acres” [13]. In the championship course case study where the 5 most recently opened courses had an average footprint of 47 acres larger than the 5 oldest courses, 260 acres to 213 acres respectively [13].

A 2017 article on physical activity accrued while playing golf in the British Medical Journal by Luscombe et al. reviewed 19 studies. Five studies had both energy expenditure and distance walked during the round of golf with using either a cart or carrying the clubs as an endpoint [8,14,15,16,17]. The articles were reviewed for course yardage and a google search was used to find the current course length. Table 2 is a summary from the BMJ article with the Energy Expenditure (EE), original course distance, distance walked during the round, and the currently published distance.

In all the studies, there is a significant discrepancy between the original course distance when the article was published and the currently published distance. This can cause an underestimation of the Energy Expenditure required to play golf competitively if fueling strategies are based on original course yardage published in older research. 

### 2.4. Hitting the Golf Ball

The energy required to hit a golf ball can be divided into the energy to swing the club and the athlete’s contribution to torque their body through the swing plane. 

Golf Laboratories manufactures robots to conduct independent testing of golf equipment for the United States Golf Association (USGA). In Table 3, the required amperages for the robot to swing the golf club at various velocities and subsequent conversion to energy in kcal/hour are listed (Personal Communication).

Using a gyroscope or magnetometer attached to the body known as Inertia Measurement Units (IMU) Kim and Park measured the average golf swing to be approximately 2 s [18]. At 115 mph, the work for the robot to swing the club is approximately 0.02 kcal. 

The athlete’s contribution was measured by two studies varying either club choice or the athlete’s experience. Outram and Wheat, using similar IMU technology to measure the total work required to swing the club, varied between 223 to 269 joules depending on the club used [19]. Nesbit and Serrano used a computer model of 4 different golfers with varying handicaps and work varied between 235 and 355 Nm with the higher work observed with the scratch golfer [20]. The 2 studies yielded comparable results. Comparing the robot data to the two swing studies, the majority of energy to hit a golf ball is utilized to rotate the body and not swing the club.

Nesbit and Serrano showed that a scratch golfer produced 355 Nm during a golf swing [20]. If the 355 Nm work produced is converted to kcal (multiplied by 0.00024) the golfer produced only 0.08 kcal per swing. If the athlete used 80 strokes to complete the round that’s only 6.4 kcal. The majority of energy utilized during a round of golf is walking the course independent of the method of club transportation.

## 3. Energy System Utilization 

### 3.1. Anerobic Contribution

The golf swing is a short intense movement lasting roughly 2 s based on the study by Kim and Park [18]. Information about anaerobic energy production during short-lasting maximal exercise is scarce [21]. Gorostiaga et al. studied 6 recreational endurance athletes that performed a set of 10 rep maximum bilateral leg presses. During the first 5 repetitions, average contraction time per repetition was 1.59 ± 0.3 s and during the second 5 repetitions, the corresponding contraction times were 20–25% longer (1.96 ± 0.35). The major reduction in Phosphocreatine (PCr) concentration occurred within the first 5 repetitions of exercise, showing that energy is primarily derived from PCr with a smaller contribution of glycolysis. During reps 6 to 10 average ATP utilization accounted for by phosphagen decreased from 54% to 19%, “ATP utilization from anaerobic glycolysis increased from 46 to 81%, muscle lactate accumulation was more substantial during the second 5 repetitions, and glycolysis was the major energy source” [21]. 

### 3.2. Aerobic Contribution

Sell et al. compared the metabolic demands of a golfer with a gas analyzer under three different conditions: Carrying clubs, pushcart, and riding in a cart. Table 4 illustrates that as the physical demand increases based on the modality of club transportation so does the oxygen demand [22].

Luscombe et al. reviewed 19 studies on the physical activity and health benefits of playing golf.

The study concluded Golf is primarily a moderate intensity physical activity:

- Fifty per cent of Metabolic Equivalent of Task (MET) values stated in the studies are within the range of moderate intensity 3–5.9.
- Values for %HRmax are within light intensity (50–63%) and moderate-intensity (64–76%) using the mean range for moderate-intensity as 101–119 bpm
- Average VO_2_ was close to the moderate threshold of 10.5–20.7 mL/kg/min.

Ramos-Jimenez et al. compared the RER in eight endurance-trained and untrained males and concluded that even at submaximal intensities “trained subjects, exercising at higher absolute workloads, showed significantly lower RER values, higher VO_2_, and lower blood lactate concentrations than untrained subjects” [23]. 

Since golf is played at submaximal exercise thresholds, endurance conditioning and fueling the glycolytic and oxidative pathways can improve performance, and athletes should not focus only on strength training and improving the Phosphocreatine (PCR) system.

## 4. Physical Characteristics

### 4.1. Age and Height

At the professional level, the event winners, top 5 and top 20 money list positions were ½ year to 1 ½ years less than the tour average of 31.6 years [24]. 

In 2018, the 10 longest hitters on the PGA tour were 1.8 cm taller than the average of 182.1 cm and 3.9 cm taller than average of 181.6 cm on the European Tour. “From 1995–2018 there has been only 1 year on the PGA Tour where the average tour height has exceeded the top 10 on the money list. On the European Tour there have been only 4 years where this has occurred” [24].

There are no statistics available at the amateur level.

### 4.2. Strength and Distance

Jack Nicklaus and Tiger Woods have won the most PGA tournaments. When Nicklaus played his drive averaged 276 yards and was the longest on tour. He was 4.5% longer than the PGA average and 2.15% longer than the rest of the top 10. In Tiger Wood’s first Masters Championship in 1997 his drive averaged 323 yards and was longer than any of his competitors, scored 18 under par, and won by 12 strokes [25,26,27]. 

The LPGA is no different. In 2020 more than 100 drives have been recorded over 300 yards through 14 events compared to less than half that number in the entire 1995 season [28].

Strokes gained are based on ShotLink data accumulated by the Professional Golf Association (PGA) Tour and are the average number of strokes during a round relative to the average Professional Golf Association (PGA) Tour tournament field. In 1997, Tiger Woods led in strokes gained with 3.20 strokes per 18 holes. 65% of the total (2.08 strokes) was attributed to his long game defined as shots over 100 yards from the hole [29]. 

Golf courses then became “Tigerproofed” by adding length, and the 2017 US Open at Erin Hills was the longest major championship playing at 7741 yards [25]. Strokes gained analysis of Professional Golf Association (PGA) Tour data shows that “the long game is the most important factor explaining the variability in professional golf scores and this accounts for more than two-thirds of the scoring differences between Professional Golf Association (PGA) Tour golfers”. From 2015 to 2018, distance was either the first or second most important predictor of success on the Professional Golf Association (PGA) Tour [29]. 

Although strength is prioritized at this level, stamina to maintain distance becomes essential with golf courses playing longer each year. Aerobic fitness cannot be completely ignored since endurance-trained athletes perform better at submaximal workloads as Ramos-Jimenez concluded [23]. 

## 5. Training 

### 5.1. General Considerations

Smith et al. reviewed 13 studies of conditioning programs to improve the fitness of golfers. All the studies utilized strength and muscular conditioning programs and all but 2 showed an increase in clubhead speed [30]. In Smith’s review, only one article “incorporated cardiovascular conditioning into the training program” [30]. With longer playing distances and longer golf courses a novel approach to incorporate aerobic training needs to be considered to reduce fatigue. 

Petre et al. studied 16 highly trained ice hockey and rugby players and subjected them to a resistance training protocol and randomized them to either additional continuous endurance training or High Intensity Interval Training (HIIT). Both groups had an increase in Time to Exhaustion VO_2_ Max. Both groups had a 12–14% increase in their lower body strength as measured by a 1 rep maximum parallel squat showing that the integration of aerobic training does not negate the effects of strength training [31].

The ratio of aerobic training compared to strength training will be dependent on the skill of the player. Less experienced players will have more errant shots due to ball dispersion and require more aerobic training since increased energy will be spent traversing the course while transporting the clubs. Experienced players will have more accuracy and need more strength training to increase driving, long shot distance, and increase strokes gained to enhance performance. Additionally, players on the Professional Golf Association (PGA) Tour have caddies for club transportation lessening their aerobic demand. 

A Framework for Periodized Nutrition for Athletics by Stellingwerf et al. divides training into macro, meso, and micro cycles depending on the athlete’s goals [32]. Utilizing this concept, training the different skill levels is covered below. 

### 5.2. High School

High school golf is the only level with a designated season with athletes playing in the spring. Training begins in the fall and depending on the winter climate may continue indoors. Macro cycles during the summer and fall can focus on building strength and more importantly increasing aerobic capacity for club carriage in these less skilled golfers.

Meso cycles could be initiated in the winter months to maintain gains and adapt the gut to utilize carbohydrate loads necessary for competition prior to, during, and recovery from training sessions. Spring would have micro cycles between tournaments to optimize fueling strategies with carbohydrates during practice rounds. 

### 5.3. College

College athletes play throughout the school year but do have the summer to support a macro cycle of 2–3 months. Depending on the skill of the college player an emphasis on either aerobic or strength training during this period can address individual deficiencies to optimize success. Distance and strokes gained become more important at this level. 

Short meso cycles can be implemented for 1–2 weeks between competitions for minor modification but need to be curtailed in time for micro cycle muscle recovery and glucose loading prior to competition.

### 5.4. Professional

Professional athletes play tournaments throughout the year and must integrate meso and micro cycles into their schedule. Major Professional Golf Association (PGA) and Ladies Professional Golf Association (LPGA) Tour championships are played from April to July. There is no option for macrocycles unless the athlete takes off a prolonged time period. Although Bryson Dechambeau added 20 pounds of muscle and increased his drive from 302 to 321 yards and won the US Open in June of 2020 using the 9-month COVID lockdown as strength focused macrocycle. 

With caddies carrying their clubs the aerobic demand is less than college athletes. Course yardage is longest, and strength needs to be prioritized to maximize strokes gained but aerobic training should be incorporated into the regimen to reduce fatigue as discussed previously.

## 6. Nutritional Strategies during Training and Competition

### 6.1. General Considerations 

Fluid and glucose management are the two nutritional issues that can affect performance during training and competition.

Total Daily Energy Expenditure can be obtained by first calculating the resting metabolic rate (RMR) which is best estimated in athletes using the Cunningham equation [33]:

RMR = 500 + 22 (FFM)

The RMR is then multiplied by an appropriate physical activity level (PAL):

Total Daily Energy Expenditure = RMR + RMR (PAL)

One round of golf utilizes approximately 1000 kcal and takes 3 to 4 h. Since golf is a “moderately active” physical activity the PAL is 1.8 utilizing the NIH Body Weight Planner [34]. If any additional exercise is performed the same day the PAL factor can be increased from 2.2 to 2.5. Carbohydrate, protein, and hydration guidelines can be implemented as described in the American College of Sports Medicine (ACSM) Joint Position Statement on Nutrition and Athletic Performance [35]. 

Modifications specifically for a round of golf and related training based on available research are listed below.

### 6.2. Carbohydrates 

Gollnick et al. did muscle biopsies for glucose on cyclists pedaling at 31% of max aerobic power (VO_2_ 6.4 kcal/min) a slightly higher level than playing golf. The biopsies showed decreased staining of both slow and fast-twitch muscle fibers (ST and FT) at variable rates. Glycogen depletion occurred first in the FT fibers with only 5% of the FT fibers moderately stained after 40 min. At 3 h only 8% of ST fibers were moderately stained for glycogen and the rest were negative.

“This glycogen loss of the ST fibers was observed at a rather early stage after the onset of exercise. This suggests that the ST fibers were heavily engaged in this rather light exercise and consumed glycogen at a rapid rate” [36]. 

In golf, slow-twitch fibers would be activated while walking the course while fast-twitch fibers would be used during the golf swing and further establishes the need for carbohydrate supplementation during competition to refuel both ST and FT fibers since golf is played at submaximal levels for several hours.

On golf days or strength training days carbohydrate needs should be 5–7 g/kg/day.
On aerobic training days, carbohydrate needs are higher at 6–10 g/kg/day.
Total daily carbohydrate needs should be between 30–45 g/kg ffm/day.
And no less than 30 kcal/kg ffm/day to prevent Relative Energy Deficiency in Sport (RED-S) especially in female athletes.

### 6.3. Fueling Strategies Prior to Training, during Training, and during Recovery of Golf

Approx. 1–4 h prior to golf or exercise carbohydrate load with 1–4 g/kg.
Golfers can snack on the course and should consume 30–60 g of carbohydrate/hour during a round of golf.
If a session of weights or endurance followed by a round of golf in the afternoon need carbohydrate recovery of 1 gm/kg/h for 4 h.
If a single session 15–25 g of carbohydrate within 1–2 h after training sessions.

### 6.4. Protein

Protein intake of 1.2 to 2.0 g/kg/d with the less skilled players at the lower limit and increasing intake as the distance becomes a priority.
Then, 15–25 g of protein within the first 2 h of completing a training session.
Protein sources should include Branched Chain Amino Acids (BCAA).

### 6.5. Hydration and Heat Acclimitization

Golf is played outdoors in warmer climates. Athletes can drink ad libitum during a round, but dehydration can affect performance if fluid intake is not adequate. Smith et al. studied 7 low handicap golfers both in the euhydrated and mildly dehydrated state. The −1.5% body weight was induced by fluid abstinence the night before and morning of testing. The dehydrated group’s shot distance decreased from 128 to 114 m, off target accuracy increased from 4.1 to 7.9 m, and the dehydrated group significantly misjudged distance compared to the euhydrated group from 8.8 to 4.1 m [37]. 

Heat acclimatization can affect hydration status causing core temperature to rise. When the body’s core temperature exceeds 39° centigrade gastrointestinal function declines affecting absorption of nutrients which decreases performance and affects the health of the athlete [38].

As exercise demand increases in warmer environments nutritional strategies to regulate body temperature in athletes have developed to increase endurance measured by time to exhaustion (TTE). Maughan and Galloway found that cyclists exercising at 60% of VO_2_ max that were not heat acclimatized, had a longer TTE, drinking more fluid. 

The TTE of a 3L/2% carbohydrate drink was 118 min compared to 1.45 L/15% carbohydrate drink of 84 min, or a control with no drink of 70.9 min. The median rectal temperature at TTE was the same despite differences in exercise time [39]. 

Siegel et al. randomized 10 males to either a cold-water drink or a slurry and ran them to exhaustion. The slurry group had a significantly prolonged TTE by 9.5 min and the TTE of the slurry group had a higher rectal temperature than the control group [40]. 

The two studies demonstrate that both the volume and temperature of ingested fluids can mitigate the negative effect of heat stress on endurance by different physiologic mechanisms.

Cheung et al. compared moderately fit men (VO_2_ max 40–50 mL/kg/min) to highly fit men (VO_2_ max > 55 mL/kg/min) and ran them to exhaustion in either a euhydrated or hypohydrated state in semipermeable protective clothing to simulate an uncompensable heat stress environment. The weight of the highly fit group averaged 76.8 kg, whereas the moderately fit group’s average weight was 92.9 kg.

The highly fit men had a greater change in rectal temperature with both a lower initial temperature and a higher final temperature and this was a major contributing factor to the longer TTE in both the euhydrated and hypohydrated trials. The study concluded that heat tolerance during exercise was improved by fitness level and TTE occurred earlier in the higher weight group regardless of hydration status [41].

Athletes should weigh themselves before and after a round of golf minus fluid and food consumed to better estimate sweat losses and individualize fluid replacement.

Consume 2–4 mL/lb in the 2 to 4 h before exercise. 

Athletes can drink ad libitum on the course but should consume 400–800 mls/h during a round of golf. 

Drinks containing sodium in a balanced electrolyte solution should be included since golf rounds last more than 2 h to avoid hyponatremia and hypohydration as outlined in the American college of Sports Medicine Joint Position Paper. 

Female athletes should be on the lower limit of replacement to avoid hypohydration and replacement should include sodium in a balanced electrolyte solution.

Recovery hydration should be 1.25–1.5 L fluid for every 1 kg of body weight lost.

## 7. Putting

### 7.1. General Considerations

Putting is the second biggest predictor of success in NCAA Division 1 male golfers [42]. The putter is the only club that is used on every hole making it the most frequent club used during a round of golf. 

### 7.2. Carbohydrate and Caffeine

Stevenson et al. evaluated 20 male golfers with an average handicap of 15. They were given a drink containing 6.4 g of carbohydrates with 100 mg of caffeine and their putting success was evaluated while walking a simulated golf course. Compared to baseline, the treatment group made more 2- and 5-m putts during the last 6 holes of the round [43]. 

### 7.3. Cricket and Pistol Shooting

Insight can also be obtained from other sports that rely on precise movements.

“Cricket is frequently played in thermally stressful environments without adequate breaks for rehydration” [2]. Seven sub elite male cricket players were recruited to bowl 6 overs (36 deliveries) both in the euhydrated and hypohydrated state which was induced by exercising in a heated room. Their length and line were then compared for accuracy. The hypohydrated state impaired bowling line accuracy by 16.4% and length by 15.4% showing decreased motor skill performance [2]. 

Conversely, Brown et al. recruited 8 police officers and evaluated shooting performance before and after exercise to volitional exhaustion at 85% their age-predicted maximum heart rate and found no difference in either their accuracy or precision; and that shooting performance was not negatively affected by fatigue [44]. 

These studies indicate that adequate hydration and carbohydrate status may be key determinants to putting success but not aerobic fitness. 

## 8. Ergogenic Supplements

### 8.1. Caffeine

As previously noted, a combined carbohydrate caffeine drink improved putting success during the last 6 holes of a round. Mumford and Tribby recruited 12 male golfers with handicaps between 3 and 10 and gave them solely 155 mg of caffeine at the start of the round and after 9 holes. In this study, no benefit was obtained with putts per round, but there was a significant improvement in iron club accuracy, driving distance and overall score [45].

### 8.2. Menthol

“Menthol has been shown to improve exercise performance in the heat by enhancing cold sensations in the mouth and inhibit the perception of warmth” [46]. Flood et al. studied 8 non-acclimated males with a 0.01% L-Menthol swill and measured their TTE at various Relative physical Exertion (RPE) levels followed by a maximal effort sprint in a heat chamber. 

The perception of cooling was more significant in the L-Menthol group with no difference in core temperature. 

TTE was also increased by 7% (23:23 and 21:44 min) and power output during the first 3 min was higher in the L-Menthol group. There was an accelerated decline in power output identified after 30% of the RPE trial was completed. Peak power during the post-RPE isokinetic sprint was lower in the menthol group (9 vs. 3.4%) [46]. 

L-Menthol has a short intense performance effect and should be utilized by golfers towards the end of the round when fatigue is most prevalent as opposed to throughout the round to prevent fatigue from occurring. 

### 8.3. Creatine

Ziegenfuss et al. evaluated the effect of a combined caffeine and creatine supplement with golfers during a 1-month strength training regimen. Twenty-seven male golfers with handicaps between 5–15 took a proprietary supplement with 5 g of creatine and 100 mg of caffeine twice a day for 2 weeks, then once a day for 2 weeks. Driving distance increased 13.9 yards (from 269.6 to 283.5) in the supplemented group [47]. 

### 8.4. Vitamin D

Golfers travel frequently. Sleeping and eating in unfamiliar environments can affect their immune system integrity. In Cohen et al. subjects with less than 7 h of sleep were 2.94 times more likely to develop a cold than those with 8 h or more of sleep after being exposed to a rhinovirus challenge [48]. 

The inability to manage food choices and reliance on processed foods “characterized by the Western Diet enhance inflammation while muting our immune system’s ability to respond to and ultimately control infections” [49]. 

Adequate Vitamin D levels have been shown to reduce the incidence of upper respiratory tract infections in Taekwondo athletes while training when baseline levels are deficient and supplemented with 5000 IU daily [50]. 

Golfers can take advantage of “The body’s ability to “synthesize Vitamin D when exposure from UVB sunlight is adequate” [51] as noted by Kryzwanski et al. which measured Vitamin D levels in elite Polish athletes in various environments. The study concluded that while the group that trained in South Africa (and Tenerife) had adequate 25(OH)D levels the group that trained outdoors in Poland was deficient by Institute of Medicine (IoM) criteria and never exceeded 30 ng/mL [52]. The therapeutic level of Vitamin D varies with its role in the body. The Institute of Medicine (IoM) Committee recommendation of 25(OH)D levels >20 ng/mL is for bone health in the general population [53]. 

Immune system integrity requires higher levels. An NHANES review of 14,108 participants found that “compared with individuals with 25OHD levels ≥30 ng/mL (75 nmol/L), those with levels <30 ng/mL had “A 58% higher adjusted odds of acute respiratory infection” [54]. 

Athletes may require even higher levels, as noted by He et al. During a 4-month period observing endurance athletes, the incidence of URTIs was inversely proportional to the Vitamin D levels. The least number of infections and shortest duration was in the optimal group with 25(OH)D levels >120 nmol/L compared to the deficient group with Vitamin D levels <30 nmol/L [55]. 

Creatine and caffeine have been shown to improve performance. 

Vitamin D can reduce the incidence of infection but levels of 25(OH)D need to be higher than the general population and exposure to sunlight may not provide adequacy and supplementation may be required. 

## 9. Conclusions

The goal of performance nutrition is to optimize the athlete’s ability to train and ultimately compete. This review has illustrated how the different energy systems are engaged during a round of golf, with the majority of energy being utilized by walking the course. Current golf courses are longer and previous assessments of energy expenditure in the literature are inadequate compared to current data available in the PGA Tour’s Distance Insight Report. Sell et al. showed that during a round of golf, as the metabolic demand increases, so does the Respiratory Exchange Ratio (RER). Ramos-Jimenez concluded that endurance trained athletes had lower RER values compared to untrained athletes during submaximal exercise. Furthermore, Gollnick et al. showed that at submaximal exercise levels similar to golf ST fibers were heavily engaged at an early stage and consumed glycogen at a rapid rate” [36]. In conclusion, improving aerobic fitness and carbohydrate utilization will minimize fatigue and increase stamina. 

During training and competition, strategies to ensure adequate hydration and using colder fluid intake will improve acclimatization. Cheung et al. concluded that aerobically trained men that weighed less were better acclimatized, tolerated heat stress better, and had a slower onset of fatigue as measured by time to exhaustion (TTE) compared to a control group. 

Distance to drive the ball should be prioritized at the higher skill levels as noted in the Distance Insight Report and increasing protein intake will complement strength training and improve driving distance as shown by Ziegenfuss et al. Endurance training can be integrated with aerobic training without sacrificing strength as shown by Petre et al. and ensuring adequate energy availability with carbohydrate supplementation during training and recovery improves strength and endurance as noted by Gollnick et al. muscle biopsy study performed at submaximal exercise thresholds similar to a round of golf. 

Ergogenic supplements can help golfers in a variety of situations. Although caffeine can improve focus its beneficial effect on putting is not conclusive, but it did increase driving distance, iron accuracy, and overall score. Menthol also has been shown to improve acclimatization and prolong time to exhaustion (TTE) but it has a short lived effect and should be used strategically. Vitamin D has been shown to improve immune system integrity in athletes which is a concern to professional golfers that travel frequently but the level needs to be higher than the current Institute of Medicine recommendations and adequate sunlight exposure may not provide these levels according to He et al. Ultimately ergogenic supplements should be tailored to the needs of the individual athlete and utilized in a manner that will maximize their effectiveness under the supervision of qualified clinicians. 

Table 5 is a summary of recommendations.

Limitations of this review include restricted article access. In Smith et al.’s Review of Conditioning Programs for Golfers [31], there was only one study that incorporated aerobic fitness into the regimen (Lennon et al.), yet this article was unobtainable despite two institutional requests. The second issue was difficulty identifying open access journals that do not use the peer review process but are featured in prominent golf magazines and news publications [56]. Lastly, there are few studies dedicated to performance nutrition and training competitively for golf, and even fewer focusing solely on women golfers. This is an area of future research desperately needed due to weight, metabolic, and anatomic differences that cannot be inferred from studies performed on men. 

## Figures and Tables

**Table 1:** Current distances for different competitive levels.

Skill Level	Gender	Distance (yds)	Distance (Meters)
High school	Boys age 15–18	6500–7100	5943–6492
	Girls age 15–18	5600–5850	5120–5349
NCAA	Men	6500–7300	5943–6675
	Women	5800–6300	5303–5760
Professional	PGA	6800–7765	6217–7100
	LPGA	6200–6600	5669–6035

NCAA-National Collegiate Athletic Association; PGA—Professional Golfers Association; LPGA—Ladies Professional Golfers Association.

**Table 2:** Luscombe et al. summary table of distances and energy expenditure.

Study	Year Published	Gender	Holes	Club Transportation	Mean Distance Walked (Km)	TEE (kcal)	Article Course Distance (m)	Current Published Distances (m)
Crowell	1970	M	9	Pull cart	4.58 +/− 0.44	411	2982 *	3108–3550 **
			9	Carry	4.02 +/− 0.52	450	2982 *	3108–3550 **
Gabellieri	2011	M	18	Carry	8.7 +/− 0.6	1202	6067 *	6217–7100
Dear	2010	M	9	Carry	4.4 +/− 0.36	511	2504	3108–3550 **
Zunzer	2013	M	18	Mixed	10.5 +/− 0.94	926	5525–5919	6217–7100
		F	18		9.89 +/− 0.81	556	4871–5307	5669–6035
		M	9		5.32 +/− 0.48	520	2762–2959	3108–3550 **
		F	9		5.25 +/− 0.16	273	2435–2653	2834–3017 **

* Listed course yardages converted to meters and divided by 2 when 9 holes played. ** PGA published yardages converted to meters and divided by 2 for 9 holes played. M: male, F: female.

**Table 3:** Golf laboratories club velocity data.

Club Velocity (mph)	Power (amps)	Energy (kcal/hour)
70	12	10.3
75	15	12.9
80	19	16.3
85	22	18.9
90	25	21.5
95	28	24.1
100	31	26.7
105	34	29.2
110	37	31.9
115	40	34.4

Conversion to kilocalories/hour by multiplying by 0.8598.

**Table 4:** Sell et al. club carriage and metabolic demands.

Transportation	Average Heart Rate (bpm)	Average VO_2_ mL/kg/min	Expired VO_2_ L/min	Respiratory Exchange Ratio (RER)
Walking	120	22.4	50.8	0.87
Pushcart	100	18.3	44.2	0.63
Riding cart	88	15.6	33.1	0.71

**Table 5:** Summary table of recommendations.

Findings/ Reccommendations	Summary	Level of Evidence
Golf course distance walked is longer than published yardage	Many factors affect the total distance including longer courses, shot dispersion, and inter-tee distance	Strong: Multiple studies reviewed pertaining to golf courses and using golfers
Walking the course utilizes the most energy and the major energy system utilized playing golf is aerobic respiration	Of the 3 causes of energy expenditure: Club transportation, hitting the ball, walking the course uses the most energy	Strong/Good: Multiple studies reviewed using golfers, but related sports used to evaluate the anaerobic contribution to swing the golf club
Aerobic training improves acclimatization and performance	Endurance trained athletes showed less fatigue than untrained counterparts and a longer time to exhaustion	Good: Study reviewed in related sports at similar VO_2_ max as playing golf
Optimizing glucose and hydration in competition increases endurance, improves performance, and improves putting	Dehydration can cause fatigue, decrease shot accuracy and distance. Golf uses FT and ST fibers that utilize glycogen at an accelerated rate at submaximal levels	Strong/Good: Dehydration and putting studies performed on golfers. Related sports used to evaluate glycogen use in muscle fibers
At higher competitive levels strength and distance become more important	Strokes gained from the long game and driving distance are key determinants of success at elite competitive levels for both men and women	Strong: Studies reviewed using golfers
Caffeine supplementation improves performance	Caffeine improves club accuracy, driving distance, and overall score but its beneficial effect on putting is debatable	Strong: Studies reviewed using golfers
Menthol and cold slushies improve acclimatization and performance in hot environments	Both Menthol and slushies reduce fatigue and increase TTE. Menthol maintained peak power for short periods. Slushies lowered core temperature	Good: studies reviewed on endurance athletes
Creatine supplementation in addition to a strength regimen improves performance	Creatine supplementation with a strength regimen increased driving distance	Strong: Study reviewed using golfers
Golfers that travel frequently are prone to infection due to alterations in diet and sleep. Vitamin D can inhibit URTIs in athletes	Vitamin D has been shown to inhibit infections but the level for athletes is higher than the current IOM recommendations	Good: Studies reviewed on endurance athletes
